# Pulsatile tinnitus due to intracranial arteriovenous fistula indirectly identified by carotid Doppler ultrasound

**DOI:** 10.1590/1677-5449.202500462

**Published:** 2026-05-08

**Authors:** André Câmara Matoso Chacon, Ricardo de Alvarenga Yoshida, Sally Tan, Samantha LaFotaine, Prakash Vishwanathan, Tulio Brasileiro Silva Pacheco, Gutenberg do Amaral Gurgel

**Affiliations:** 1 AngioVascular, Natal, RN, Brasil.; 2 Universidade Estadual Paulista Júlio de Mesquita Filho – UNESP, Faculdade de Medicina de Botucatu, Botucatu, SP, Brasil.; 3 New York University – NYU, Grossman Long Island School of Medicine, Mineola, NY, USA.

**Keywords:** pulsatile tinnitus, arteriovenous fistula, carotid duplex, embolization

## Abstract

Pulsatile tinnitus is a specific type of sound caused by vascular anomalies, usually associated with turbulent blood flow, secondary to high-output stenosis or arterial tortuosity. These alterations can produce rhythmic sounds that compromise patients’ quality of life. This report describes the case of a 51-year-old woman with pulsating tinnitus in her right ear for 18 months. No significant changes were identified on arterial computed tomography angiography or cranial magnetic resonance angiography due to the non-completion of the joint venous phase. Carotid Doppler ultrasound showed abnormal flow in the right external carotid artery, with a decreased resistance index, suggesting an arteriovenous fistula. Cerebral angiography confirmed the diagnosis, showing communication between external carotid artery branches and intracranial veins. The patient underwent endovascular treatment with embolization, resulting in complete resolution of symptoms and normalization of Doppler parameters at 24-month follow-up.

## INTRODUCTION

Tinnitus is characterized as the conscious and involuntary perception of an unwanted sound in the ear, and it affects approximately 30% of the population.^1^ The auditory perception of rhythmic noise synchronized with the heartbeat is defined as pulsatile tinnitus and may be broadly classified into vascular and nonvascular etiologies. Less than 10% of patients with tinnitus suffer from pulsatile tinnitus, which is mostly unilateral; however, in vascular pathologies, it tends to be bilateral.^2^ The most common cause of intermittent pulsatile tinnitus is uncontrolled systemic hypertension,^1^ so blood pressure control is usually the first intervention.

An intracranial arteriovenous fistula (AVF) consists of abnormal communications between dural branches of extracranial arteries and intracranial venous sinuses or meningeal veins. In addition to tinnitus, patients with AVF may present with intracranial hemorrhage and cerebral venous infarction. Initial diagnosis of intracranial AVF requires cranial computed tomography angiography (CTA) or magnetic resonance imaging (MRI).^1^

Carotid Doppler ultrasound (CDU) is a simple and practical method for screening patients with pulsatile tinnitus and is useful for detecting hemodynamic changes in the external carotid artery (ECA) and the occipital artery.^3^

## CASE DESCRIPTION

A 51-year-old woman presented with complaints of headache and right-sided pulsatile tinnitus for 18 months. She was evaluated by a cardiologist and an otorhinolaryngologist and underwent arterial CTA and venous CTA separately, as well as intracranial MRI, without specific findings. She was prescribed bisoprolol and ramipril, with partial symptom improvement.

**Figure 1:**
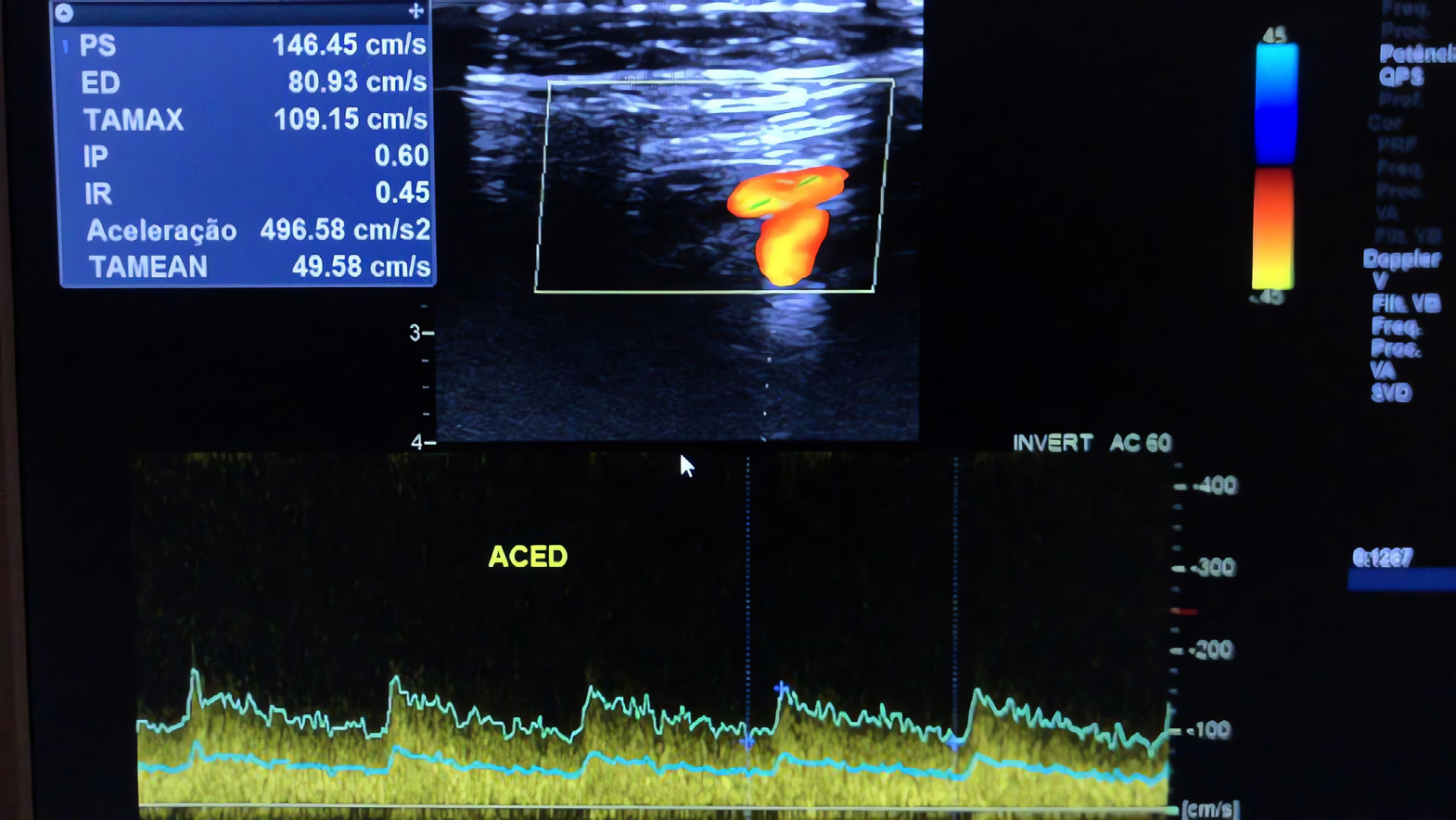
External carotid artery waveform with low resistance index.

**Figure 2:**
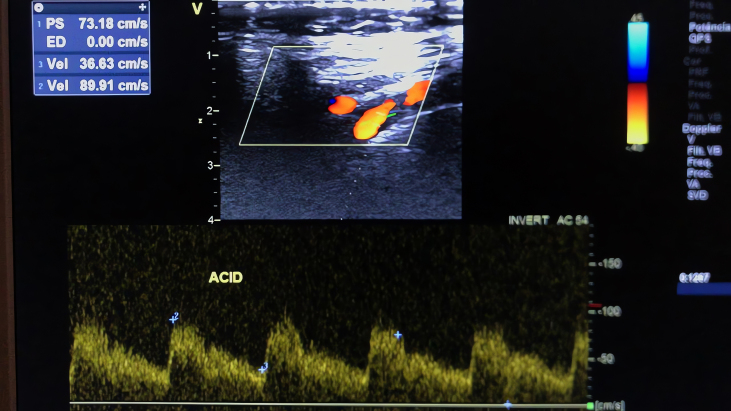
Internal carotid artery waveform with normal flow pattern for comparison.

Subsequently, the patient underwent a CDU, which found an unusual waveform pattern in the ECA, with a very low resistance index (RI) (0.45) (Figure 1), comparable to that of the internal carotid artery (0.59) (Figure 2). The RI is defined as (peak systolic velocity − end-diastolic velocity)/peak systolic velocity. This value was considerably lower than that of the internal carotid artery, suggesting the presence of an AVF.

**Figure 3:**
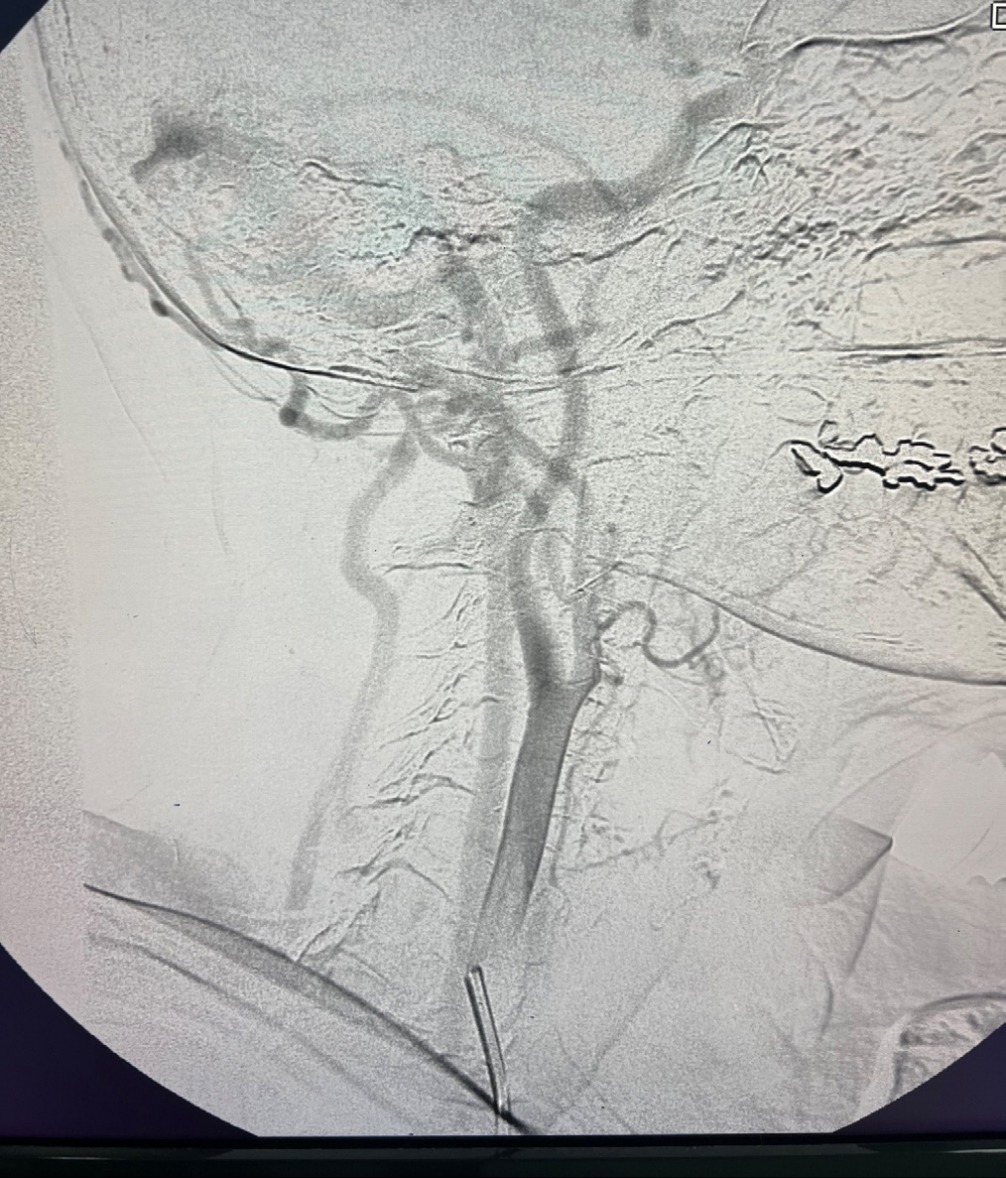
Cerebral angiography showing arteriovenous communication between a branch of the external carotid artery and the transverse sinus.

**Figure 4:**
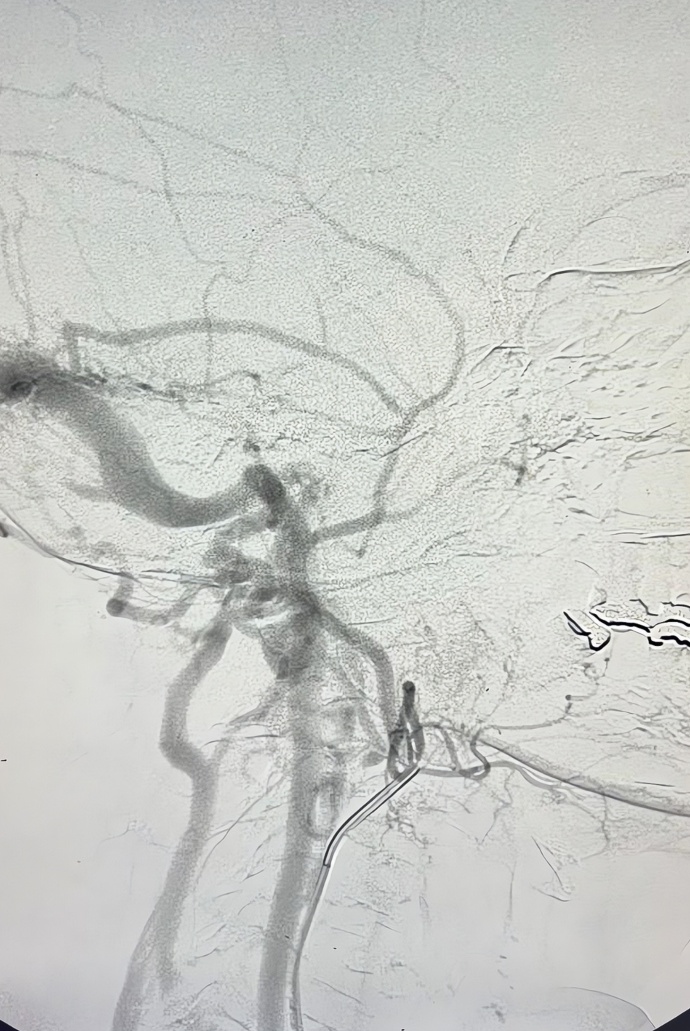
Early venous drainage into the right internal jugular vein.

Given this finding, the patient underwent cerebral angiography, which identified fistulas connecting branches of the ECA to an intracranial vein (transverse sinus, Figure 3), with rapid drainage into the right internal jugular vein (Figure 4), corroborating the patient’s symptoms.

**Figure 5:**
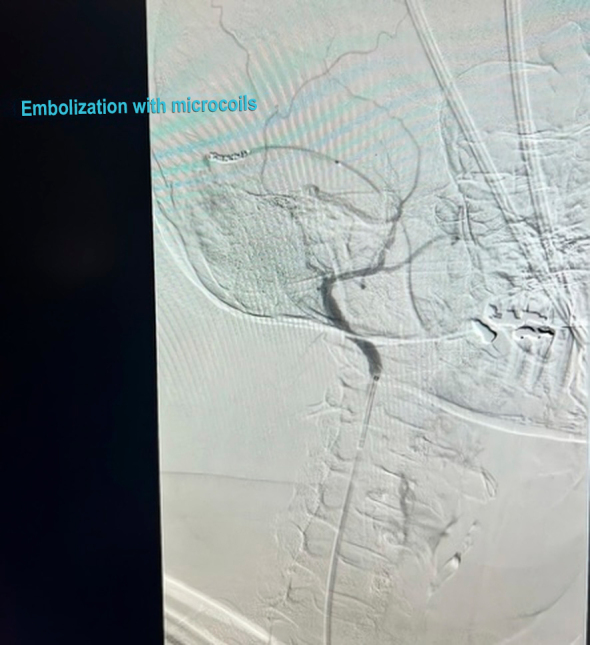
Angiographic image after embolization, showing reduction of fistulous flow.

**Figure 6:**
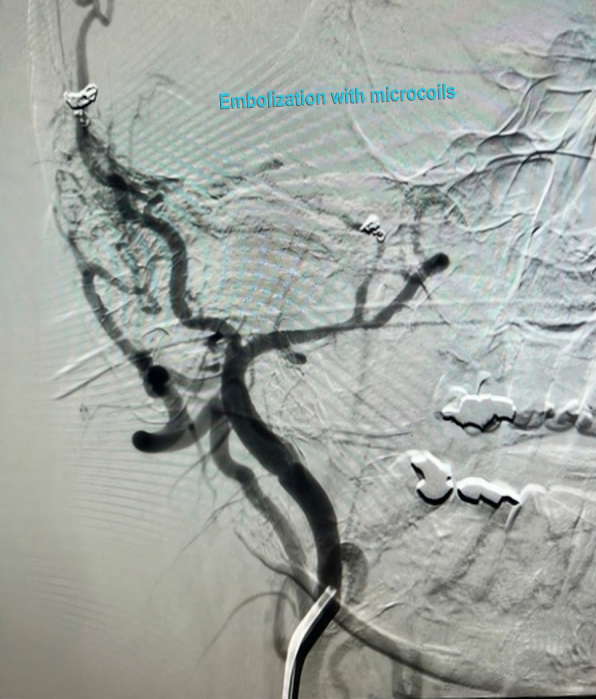
Follow-up angiography showing reduction of the arteriovenous shunt.

Subsequently, the patient underwent embolization of the main AVF communication points through selective catheterization of the right ECA and implantation of six detachable microcoils (Concerto): one 2 mm × 4 cm unit, four 2 mm × 6 cm units, and one 2 mm × 8 cm unit. Follow-up angiography found a significant reduction of the arteriovenous communication (Figures 5 and 6).

**Figure 7:**
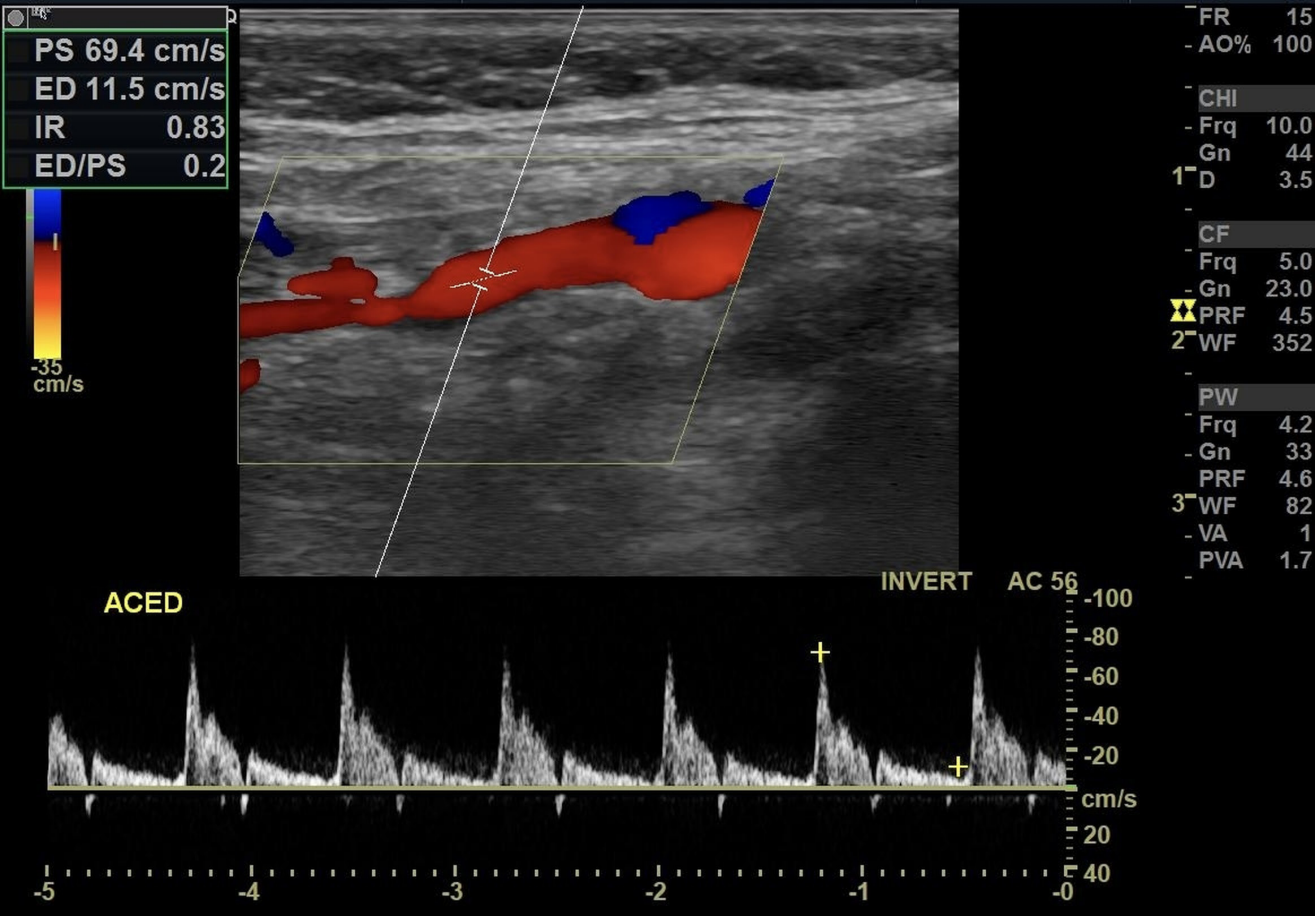
Right carotid Doppler ultrasound at post-embolization follow-up showing elevated resistance index in the external carotid artery.

On postoperative day 1, the patient reported marked improvement in tinnitus and headache. At the first outpatient follow-up visit, she was already asymptomatic. On a subsequent follow-up visit, a CDU found an increase in RI in the right ECA (preoperative: 0.45 vs. postoperative: 0.83). The patient has remained under follow-up for 24 months and is asymptomatic. CDU continues to find an elevated RI in the right ECA (Figure 7), with complete remission of symptoms and no need for antihypertensive or analgesic medication.

This case report was approved by the Research Ethics Committee at Universidade Potiguar (UNP), with Consolidated Opinion number 7.736.474 and Ethics Appraisal Submission Certificate 90441425.6.0000.5296, as determined by Resolution no. 166/2018 of the Brazilian National Research Ethics Committee.

## DISCUSSION

Pulsatile tinnitus is a multifactorial condition. The most frequent etiologies include carotid stenosis and cerebral AVFs, accounting for approximately 5%-8% and 15%-20% of cases, respectively.^4^ Both conditions may lead to stroke, justifying the importance of early diagnosis and treatment.

A detailed medical history and thorough physical examination are crucial to clarify the underlying cause of pulsatile tinnitus. It is important to investigate neurological deficits, vertigo, hearing loss, or otalgia. Otologic examination and audiometric evaluation may assist in excluding masses on the tympanic membrane, in addition to differentiating conductive from sensorineural hearing loss.^5^ Nonvascular etiologies can be ruled out through clinical assessment and initial auditory and otologic examinations. Signs such as bilateral pulsatile tinnitus, a bruit, or symptom improvement with ipsilateral cervical compression favor suspicion of vascular etiology.^6^ In this context, imaging studies try to identify neoplasms and vascular malformations. In the study by Sonmez et al.^7^ imaging examinations (ultrasound, CT, MRI) were successful in detecting the etiology of pulsatile tinnitus in 67.6% of patients.

Several studies have confirmed the usefulness of CDU as a screening method. According to Tsai et al.,^8^ an RI < 0.7 in the ECA demonstrates a sensitivity of 80.8% and specificity of 100%; in comparison, the gold standard (MRI/magnetic resonance angiography [MRA] and catheter angiography or hybrid CT angiography) achieves a sensitivity of 93% and specificity of 98%.^9^ However, it should be noted that, in addition to intracranial AVFs/arteriovenous malformations, other causes of a low-resistance pattern in the ECA on Doppler include hypervascularized tumors (such as paragangliomas), inflammatory or infectious processes in the head and neck region, and surgical or traumatic vascular shunts. In the present case, however, the absence of clinical or radiological signs suggestive of these conditions strengthened the diagnostic hypothesis of AVF, subsequently confirmed by angiography.

CDU is a noninvasive and low-cost method. Care Operative data show that 3/4 of patients with normal CDU findings also present normal results on MRA or CT angiography.^10^ If all individuals with pulsatile tinnitus were initially submitted to CDU, and only those with stenosis or abnormal hemodynamic changes were referred for MRA or CT angiography, the projected total cost would be US$ 84,407, compared with US$ 154,070 if all patients underwent MRA or CT angiography directly.

In recent years, dynamic CT angiography has gained prominence as a noninvasive modality for assessing the hemodynamics of arteriovenous lesions and assisting in diagnosis and therapeutic planning. This approach may represent a promising advance in the diagnosis and treatment of intracranial AVFs.

Pulsatile tinnitus secondary to AVF may be associated with severe complications, such as intracranial hemorrhage and cerebral venous infarction, justifying the need for detailed clinical and imaging investigation. As illustrated in this report, CDU may be used for the initial screening, followed by diagnostic confirmation (MRI or CT) only in select cases with abnormal CDU findings, and subsequent minimally invasive endovascular treatment with microcoils, potentially reducing costs.

AVF treatment is indicated both for symptomatic relief of pulsatile tinnitus and for mitigation of stroke risk. Narsinh et al.^11^ proposed a management algorithm for vascular causes of pulsatile tinnitus based on the degree of stroke risk associated with the lesion. With current advances, most diagnosed intracranial AVFs can be successfully treated via an endovascular procedure, as observed in this case.^12^

## Data Availability

Os dados que sustentam este estudo estão disponíveis mediante solicitação ao autor correspondente, ACMC, pois envolvem informações clínicas sensíveis do paciente.
