# Supplementary material for: Pulsatile tinnitus due to intracranial arteriovenous fistula indirectly identified by carotid Doppler ultrasound
Source: J Vasc Bras. 2026 May 8;25:e20250046. doi: 10.1590/1677-5449.202500462 (PMC13193368; doi:10.1590/1677-5449.202500462)
Supplement: Translation

# Translation: Zumbido pulsátil causado por fístula arteriovenosa intracraniana, diagnosticada indiretamente por ultrassom com Doppler das carótidas

**DOI:** 10.1590/1677-5449.202500461

**Authors:** André Câmara Matoso Chacon, Ricardo de Alvarenga Yoshida, Sally Tan, Samantha LaFotaine, Prakash Vishwanathan, Tulio Brasileiro Silva Pacheco, Gutenberg do Amaral Gurgel

**Affiliations:** 1 AngioVascular, Natal, RN, Brasil.; 2 Universidade Estadual Paulista Júlio de Mesquita Filho – UNESP, Faculdade de Medicina de Botucatu, Botucatu, SP, Brasil.; 3 New York University – NYU, Grossman Long Island School of Medicine, Mineola, NY, USA.

**Keywords:** zumbido pulsátil, fístula arteriovenosa, doppler de carótidas, embolização

## Abstract

O zumbido pulsátil é um tipo específico de som causado por anomalias vasculares, geralmente associadas a fluxo sanguíneo turbulento, secundário a estenoses de alto débito ou a tortuosidades arteriais. Essas alterações podem gerar ruídos rítmicos que comprometem a qualidade de vida dos pacientes. Descrevemos o caso de uma mulher de 51 anos com zumbido pulsátil no ouvido direito há 18 meses. Não foram identificadas alterações significativas nos exames de angiotomografia arterial e de angiorressonância magnética de crânio devido a não realização da fase venosa conjunta. O ultrassom com Doppler das carótidas evidenciou fluxo anômalo na artéria carótida externa direita, com índice de resistência diminuído, sugerindo fístula arteriovenosa. A angiografia cerebral confirmou o diagnóstico, identificando comunicação entre ramos da carótida externa e veias intracranianas. A paciente foi submetida a tratamento endovascular por embolização, com resolução completa dos sintomas e normalização dos parâmetros ao Doppler no seguimento de 24 meses.

## INTRODUÇÃO

O zumbido caracteriza-se pela percepção consciente e involuntária de som indesejado no ouvido, acometendo cerca de 30% da população^1^ . A percepção auditiva de ruído rítmico sincronizado ao batimento cardíaco é definida como zumbido pulsátil, podendo ser amplamente classificado em etiologias vasculares e não vasculares. Menos de 10% dos casos de zumbido são pulsáteis, sendo a maioria unilateral; entretanto, em patologias vasculares, pode a ser bilateral^2^ . A causa mais comum de zumbido pulsátil intermitente é a hipertensão arterial sistêmica não controlada^1^ , de modo que o controle pressórico costuma ser a primeira intervenção.

Uma fístula arteriovenosa (FAV) intracraniana consiste em comunicações anormais entre ramos durais de artérias extracranianas e seios venosos intracranianos ou veias meníngeas. Além do zumbido, pacientes com FAV podem apresentar hemorragia intracraniana e infarto venoso cerebral. O diagnóstico inicial de FAV intracraniana requer angiotomografia computadorizada (angio-TC) de crânio ou ressonância magnética (RM)^1^ .

O ultrassom com Doppler das carótidas (UDC) é um método simples e prático para a triagem de pacientes com zumbido pulsátil, sendo útil na detecção de alterações hemodinâmicas na artéria carótida externa (ACE) e na artéria occipital^3^ .

## DESCRIÇÃO DO CASO

Uma paciente de 51 anos apresentava queixas de cefaleia e zumbido pulsátil à direita havia 18 meses. Foi avaliada por cardiologista e otorrinolaringologista, sendo realizadas angio-TC arterial e angio-TC venosa separadamente, além de RM intracraniana, sem achados específicos. Recebeu prescrição de bisoprolol e ramipril, obtendo melhora parcial dos sintomas.

Subsequentemente, foi realizado UDC, no qual se observou um padrão de onda incomum na ACE, com índice de resistência (IR) muito baixo (0,45) (Figura 1), comparável ao da artéria carótida interna (0,59) (Figura 2). O IR é definido como (velocidade sistólica de pico − velocidade diastólica final)/velocidade sistólica de pico. Esse valor mostrava-se consideravelmente mais baixo em comparação ao da artéria carótida interna, sugerindo a presença de FAV.

**Figura 1:** Traçado da artéria carótida externa com índice de resistência baixo.

**Figura 2:** Traçado da artéria carótida interna com padrão de fluxo normal para comparação.

Diante disso, optou-se por angiografia cerebral, que identificou fístulas conectando ramos da ACE a uma veia intracraniana (seio transverso, Figura 3), com drenagem rápida para a veia jugular interna direita (Figura 4), corroborando os sintomas da paciente.

**Figura 3:** Angiografia cerebral evidenciando comunicação arteriovenosa entre ramo da artéria carótida externa e seio transverso.

**Figura 4:** Drenagem venosa precoce para veia jugular interna direita.

Posteriormente, a paciente foi submetida a embolização dos principais pontos de comunicação da FAV, por meio de cateterização seletiva da ACE direita e implante de seis micromolas (Concerto) de liberação controlada: uma unidade de 2 mm × 4 cm, quatro unidades de 2 mm × 6 cm e uma unidade de 2 mm × 8 cm. A angiografia de controle evidenciou redução significativa da comunicação arteriovenosa (Figuras 5 e 6).

**Figura 5:** Imagem angiográfica após embolização, com redução do fluxo fistuloso.

**Figura 6:** Controle angiográfico evidenciando diminuição do *shunt* arteriovenoso.

No primeiro dia pós-operatório, a paciente referiu melhora importante do zumbido e da cefaleia. No primeiro retorno ambulatorial, já se encontrava assintomática. Em controle subsequente, o UDC evidenciou aumento do IR na ACE direita (pré-operatório: 0,45 vs. pós-operatório: 0,83). A paciente permanece em seguimento há 24 meses, assintomática. O UDC continua demonstrando IR elevado na ACE direita (Figura 7), com remissão completa dos sintomas e sem necessidade de medicação anti-hipertensiva ou analgésica.

**Figura 7:** Doppler de carótida direita no controle pós-embolização, com índice de resistência elevado na artéria carótida externa.

Este relato de caso foi aprovado pelo Comitê de Ética em Pesquisa da Universidade Potiguar, sob o número do parecer consubstanciado 7.736.474 e CAAE 90441425.6.0000.5296, conforme determina a Resolução nº 166/2018 da CONEP.

## DISCUSSÃO

O zumbido pulsátil é um quadro multifatorial, cujas etiologias mais frequentes incluem estenose carotídea e FAVs cerebrais, responsáveis por cerca de 5-8% e 15-20% dos casos, respectivamente^4^ . Ambas as condições podem causar acidente vascular cerebral (AVC), justificando a importância do diagnóstico e tratamento precoces.

Anamnese detalhada e exame físico minucioso são cruciais para esclarecer a causa subjacente do zumbido pulsátil. É importante investigar déficits neurológicos, vertigem, perda auditiva ou otalgia. O exame otológico e a audiometria podem auxiliar na exclusão de massas na membrana timpânica, além de diferenciar hipoacusia condutiva de neurossensorial^5^ . Etiologias não vasculares podem ser descartadas a partir de avaliação clínica e exames auditivos e otológicos iniciais. Sinais como zumbido pulsátil bilateral, sopro ou melhora dos sintomas com compressão cervical ipsilateral favorecem a suspeita de etiologia vascular^6^ . Nesse contexto, a propedêutica por imagem busca identificar neoplasias e malformações vasculares. No estudo de Sonmez et al.^7^ , constatou-se que exames de imagem (ultrassom, TC, RM) foram bem-sucedidos em detectar a etiologia do zumbido pulsátil em 67,6% dos pacientes.

A utilidade do UDC como método de triagem foi confirmada em diversos estudos. Segundo Tsai et al.^8^ , um IR < 0,7 na ACE apresenta sensibilidade de 80,8% e especificidade de 100%; em comparação, o padrão-ouro (RM/angiografia por RM [angio-RM] e angiografia por cateter ou angio-TC híbrida) atinge sensibilidade de 93% e especificidade de 98%^9^ . No entanto, deve-se lembrar que, além das FAVs/malformações arteriovenosas intracranianas, outras causas de padrão de baixa resistência no Doppler da ACE incluem tumores hipervascularizados (como paragangliomas), processos inflamatórios ou infecciosos na região da cabeça e pescoço e *shunts* vasculares cirúrgicos ou traumáticos. Contudo, no presente caso, a ausência de sinais clínicos ou radiológicos sugestivos dessas condições fortaleceu a hipótese diagnóstica de FAV, posteriormente confirmada por angiografia.

O UDC é um método não invasivo e de baixo custo. Dados da Care Operative mostram que 3/4 dos pacientes com UDC normal também apresentam exames normais em angio-RM ou angio-TC^10^ . Caso todos os indivíduos com zumbido pulsátil fossem inicialmente submetidos ao UDC, e apenas aqueles com estenose ou alterações hemodinâmicas anormais fossem encaminhados para angio-RM ou angio-TC, o custo total projetado seria de US$ 84.407, em comparação a US$ 154.070 se todos realizassem diretamente angio-RM ou angio-TC.

Nos últimos anos, a angio-TC dinâmica tem ganhado destaque como modalidade não invasiva para avaliar a hemodinâmica de lesões arteriovenosas e auxiliar no diagnóstico e planejamento terapêutico. Essa abordagem pode constituir um avanço promissor no diagnóstico e tratamento das FAVs intracranianas.

O zumbido pulsátil decorrente de FAV pode associar-se a complicações graves, como hemorragia intracraniana e infarto venoso cerebral, justificando a necessidade de investigação clínica e por imagem detalhadas. Como ilustrado neste relato, o UDC pode constituir a triagem inicial, seguida por confirmação diagnóstica (RM ou TC) somente em casos selecionados, nos que há alterações no UDC, e posterior tratamento endovascular minimamente invasivo com micromolas, resultando em potencial redução de custos.

O tratamento da FAV é indicado tanto para alívio do sintoma do zumbido pulsátil quanto para mitigar o risco de AVC. Narsinh et al.^11^ propuseram um algoritmo de manejo para causas vasculares de zumbido pulsátil, baseando-se no grau de risco de AVC associado à lesão. Com os avanços atuais, a maioria das FAVs intracranianas diagnosticadas pode ser tratada com sucesso por via endovascular, conforme observado neste caso^12^ .

## Data Availability

The data that supports this study are available from the corresponding author (ACMC) upon request, as they involve sensitive patient clinical information.
